# Serum Neuropeptide Y Levels Are Associated with TNF-*α* Levels and Disease Activity in Rheumatoid Arthritis

**DOI:** 10.1155/2020/8982163

**Published:** 2020-04-16

**Authors:** Melissa Ramirez-Villafaña, Ana M. Saldaña-Cruz, Javier A. Aceves-Aceves, Edsaul E. Perez-Guerrero, Nicté S. Fajardo-Robledo, Edy D. Rubio-Arellano, Cesar A. Nava-Valdivia, Maria O. Carrillo-Escalante, Sylvia E. Totsuka-Sutto, David Cardona-Müller, Betsabe Contreras-Haro, Mario Salazar-Paramo, Ernesto G. Cardona-Muñoz, M. Huerta, Jorge I. Gamez-Nava, Norma A. Rodriguez-Jimenez, Laura Gonzalez-Lopez

**Affiliations:** ^1^Programa de Doctorado en Ciencias Médicas, Universidad de Colima, 28040 Colima, Colima, Mexico; ^2^Unidad de Investigación Biomédica 02, Hospital de Especialidades, Centro Médico Nacional de Occidente (CMNO), Instituto Mexicano del Seguro Social (IMSS), 44340 Guadalajara, Jalisco, Mexico; ^3^Departamento de Fisiología, CUCS, Universidad de Guadalajara, 44340 Guadalajara, Jalisco, Mexico; ^4^Instituto de Terapeutica Experimental y Clínica, Centro Universitario de Ciencias de la Salud (CUCS), Universidad de Guadalajara (UdeG), 44340 Guadalajara, Jalisco, Mexico; ^5^Programa de Doctorado en Farmacología, CUCS, Universidad de Guadalajara, 44340 Guadalajara, Jalisco, Mexico; ^6^Instituto de Investigación en Ciencias Biomédicas, CUCS, UdeG, 44340 Guadalajara, Jalisco, Mexico; ^7^Laboratorio de Investigación y Desarrollo Farmacéutico, Centro Universitario de Ciencias Exactas e Ingenierías, Universidad de Guadalajara, 44430 Guadalajara, Jalisco, Mexico; ^8^Departamento de Microbiología y Patología, CUCS, Universidad de Guadalajara, 44340 Guadalajara, Jalisco, Mexico; ^9^Departamento de Ciencias Biomédicas, Centro Universitario de Tonalá, Universidad de Guadalajara, 45425 Tonalá, Jalisco, Mexico; ^10^Centro Universitario de Investigaciones Biomédicas, Universidad de Colima, 28040 Colima, Colima, Mexico; ^11^Programa de Doctorado en Salud Publica, CUCS, Universidad de Guadalajara, 44340 Guadalajara, Jalisco, Mexico; ^12^Departamento de Medicina Interna-Reumatología, Hospital General Regional 110, Instituto Mexicano del Seguro Social, 44716 Guadalajara, Jalisco, Mexico

## Abstract

**Background:**

Neuropeptide Y (NPY) is a sympathetic neurotransmitter with effects on the regulation of inflammatory cells. The role of NPY on autoimmune inflammatory diseases such as rheumatoid arthritis (RA) is not completely understood. Therefore, we evaluate if NPY levels are markers of disease activity in RA and if there is a correlation between NPY levels and tumor necrosis factor-alpha (TNF-*α*), leptin, and interleukin 6 (IL-6) levels.

**Methods:**

Cross-sectional design, including 108 women with RA. We assessed disease activity by DAS28-ESR (considering active disease a score of ≥2.6). Serum NPY levels and anti-CCP2 antibody, TNF-*α*, IL-6, and leptin levels were quantified (ELISA).

**Results:**

Sixty-eight RA had an active disease (RA-active), and 40 were in remission (RA-remission). RA-active patients had higher NPY levels vs. RA-remission (22.8 ± 13.6 vs. 17.8 ± 10.3; *p* = 0.04). NPY levels correlated with increased TNF-*α* levels (*r* = 0.32, *p* = 0.001). Leptin or IL-6 did not correlate with NPY levels. In the logistic regression analysis, NPY increased the risk of disease activity (OR: 1.04, 95% CI 1.006-1.09, and *p* = 0.03).

**Conclusion:**

Higher NPY levels are an independent marker of disease activity in RA. This study encourages the quantification of NPY levels as a surrogate marker for RA-active. Future studies evaluating the role of NPY levels interacting with other proinflammatory cytokines are required.

## 1. Introduction

The rheumatoid arthritis (RA) is a systemic inflammatory disease affecting synovial joints leading to pannus with joint destruction and functional disability [1]. The aims of the treatment for rheumatoid arthritis include maintaining low disease activity or remission and improving pain, fatigue, inflammation, labour capacity, and health-related quality of life [2, 3]. Nevertheless, there is a high frequency of failure to conventional treatments, particularly to monotherapy with synthetic disease controlling antirheumatic drugs (syntDMARDs) [4]. Although the persistence of chronic inflammation in RA has multifactorial pathogenesis; to date, there is new evidence about the participation of the sympathetic nervous system on the regulation of inflammation in RA [5]. In this context, neuropeptide Y (NPY), a sympathetic neurotransmitter, might mediate effects on cardiovascular functions, hypertension, obesity [6, 7], and regulation of inflammatory cells [5, 8]. NPY also has a role in the link between the immune system and the neuroendocrine system [9]. In experimental studies, TNF activated the neuronal NPY promoter. In knockout NPY−/− enteric neurons from mice, a lower secretion of TNF compared to wild-type mice has been demonstrated [10]. NPY can induce the activation of immune cell response including macrophages, neutrophils, and lymphocytes inducing the release of proinflammatory cytokines including TNF-*α* or interleukin 6 (IL-6) [9].

Several reports have demonstrated abnormal concentrations of NPY in systemic lupus erythematosus and RA [11, 12]. Although studies have been performed in RA patients, the findings have shown discordant results about NPY levels [11–14]. For instance, Härle et al. identified higher NPY levels in RA patients compared to those seen in healthy subjects, but there was no association observed between NPY levels and clinical characteristics of RA patients [12]. Härle et al. identified higher concentrations of RA and SLE compared to controls, although Härle et al. found no correlation between NPY levels and disease activity in RA patients [11]. Vlcek et al. identified that the NPY levels did not differ in RA patients and controls [14].

To date, this lack of consistency between the results of the studies referred above implies that the assessment of the role of NPY levels as a possible marker of active disease in RA patients should be assessed in studies with a multivariate approach controlling potential confounders including the serum levels of other proinflammatory cytokines and leptin. Therefore, this study is aimed at determining whether NPY levels are markers of disease activity in RA and if there is a correlation between NPY levels and TNF-*α* levels.

## 2. Materials and Methods

### 2.1. Study Design

This study is a cross-sectional study.

### 2.2. Study Population

This study included 108 women with RA who were from an outpatient clinic of a secondary care centre in Guadalajara, Mexico. Selected patients were women aged ≥18 years that met the 1987 American College of Rheumatology (ACR) criteria for RA [15] and signed a voluntary consent form for the study.

In this study, we excluded patients with other autoimmune diseases, including overlapping syndrome, acute or chronic infections such as hepatitis B or C, human immunodeficiency virus, or tuberculosis. We excluded also patients with a diagnosis of cancer, chronic kidney disease, or an increase in serum transaminase levels of >2-fold of normal values. Pregnant or breastfeeding patients were also excluded from the study.

### 2.3. Ethics and Consent

The study protocol was performed according to the guidelines of the 64th Declaration of Helsinki. The Research and Ethics Committee of the Hospital General Regional #110, IMSS, in Guadalajara, Mexico, approved the study protocol under the registration number R-2014-1303-19.

All participants in this study were asked to sign a voluntary informed consent before the study inclusion. This voluntary informed consent was also approved by the Research and Ethics Board of the hospital. This voluntary consent form was according to the ethical practices in research studies following the lineaments of the Helsinki Declaration.

### 2.4. Study Development

Patients were assessed by trained researchers with a structured interview, physical examination including the clinimetrics of the disease, and laboratory studies.

### 2.5. Assessment of Disease Activity

We used for the evaluation of disease activity the Disease Activity Score for 28 joints with Erythrocyte Sedimentation Rate (DAS28-ESR) as acute phase reactant [16]. DAS28-ESR is a widely validated index accepted worldwide. This index is used as criteria of the intensity of disease activity in RA patients. We classified the RA patients into the following two groups: (a) patients with active disease (DAS28-ESR score of ≥2.6 (RA-active)) and (b) RA patients in clinical remission (DAS28‐ESR < 2.6) [17]. Additionally, we evaluated physical functioning using the validated Spanish version of the Health Assessment Questionnaire-Disability Index (HAQ-DI) [18].

### 2.6. Anthropometric Measurements

Body weight was measured using bioelectrical impedance (Tanita™), following the standardised protocols. Height was measured using a wall stadiometer (Seca™ model 206). Body Mass Index (BMI) was calculated in kg/m^2^ and classified per the parameters described by the World Health Organization (WHO) as follows: normal weight (range from 18.5 to 24.9 kg/m^2^), overweight (ranging from 25 to 29.9 kg/m^2^), and obesity (≥30 kg/m^2^) [19].

### 2.7. Body Composition Measurements Using Densitometry

Body composition was assessed using Dual-energy X-ray Absorptiometry (DXA) (LUNAR 2000, Prodigy Advance; General Electric™, Madison, WI, USA) using standardised protocols described by the manufacturer. We obtained by DXA the following parameters: fat mass (%) and lean mass (%).

### 2.8. Laboratory Determinations

An 8 h fasting venous blood sample was obtained from the RA patients and controls. From this blood sample, the serum was separated and it was stored at –20°C for NPY determinations. Levels of serum rheumatoid factor (RF) and anticyclic citrullinated peptide (anti-CCP) antibodies of second generation were also quantified. Rheumatoid factor (RF) was measured in by nephelometry in 73 patients at the time of the study, whereas anti-CCP was quantified by ELISA in 106 patients using a commercial kit (EUROIMMUN, Lübeck Germany).

### 2.9. Determination of NPY Levels and Leptin, Interleukin 6, and TNF-*α* Levels

Serum NPY levels were determined by ELISA using a commercial kit (EMD Millipore™; MI, USA). The detection range of NPY levels varies from 2 to 1,000 pg/mL. TNF-*α* level was determined in 106 patients, interleukin 6 level was determined in 89 patients, and leptin level was determined in 91 patients. All these molecules were quantified by ELISA using commercial kits (R&D Systems, Inc., Minneapolis, MN, USA). All the laboratory determinations were performed by researchers blinded to the characteristics of the patients.

### 2.10. Statistical Analysis

Quantitative variables were expressed as means and standard deviations (SD) and qualitative variables as frequencies and percentages (%). We used Pearson's correlation tests to identify a correlation between NPY levels and age, duration of RA, BMI, fat mass, and serum levels of TNF-*α*, IL-6, leptin, RF, and anti-CCP antibodies. Comparisons of proportions between groups were computed using chi-squared tests (or Fisher's exact test if required). Comparisons of means between RA and control groups were calculated using independent-sample Student's *t*-tests; a similar statistical approach was used to compare means in NPY levels between the RA-active and RA-remission groups.

Multivariate linear and logistic regression analyses were performed to adjust the variables associated with the dependent variables by confounders. Covariates included in these models were those with biological plausibility to modify according to the model the following dependent variables: disease activity and serum levels of NPY. Also, we included in the models those variables with *p* value ≤ 0.20 in the bivariate analysis. A logistic regression model was built to identify if NPY levels were associated with disease activity adjusting by other variables. Variables included in the adjustment in the final model were as follows: age, BMI, body fat mass, biologic-DMARD use (anti-TNF-*α* agents), HAQ-DI score, and NPY serum levels. In this model, we used DAS28‐ESR ≥ 2.6 (RA-active) as the dependent variable, and the forward conditional method was used to adjust confounder variables.

Multivariate linear regression analyses were performed to adjust the variables associated with serum NPY levels as the dependent variable. Age, BMI, anti-CCP levels, TNF-*α* levels, leptin levels, lean mass (%), fat mass (%), and RA disease duration were used as covariates. All the analyses were made using SPSS statistical software (IBM SPSS Statistics for Windows, Version 25.0. Armonk, NY: IBM Corp.).

## 3. Results and Discussion

### 3.1. Results

Table 1 presents a description of the clinical characteristics of the 108 patients with RA included in the study. These patients had a mean age of 58.7 years and mean disease duration of 13.9 years, and 63% of the RA patients had active disease. The following comorbidities were observed in RA patients: 70% overweight or obesity, 97.2% body fat mass of >33%, 75.9% dyslipidaemia, 39.8% hypertension, and 11.1% diabetes mellitus (data not shown in tables).

Most of the RA patients (97.2%) received synthetic-disease-modifying antirheumatic drugs (DMARDs), and 12% of the patients used biologic-DMARDs. The frequency of corticosteroid use was 81.5%, and the mean dose was 4.8 ± 3.2 mg/day. At the time of the study, 13% of patients used biologic-DMARDs: 8 (7.4%) used etanercept, 3 (2.8%) infliximab, 1 (0.9%) adalimumab, and 1 (0.9%) rituximab. None of the RA patients included in the study were receiving tocilizumab or abatacept (data not shown in table).

In the data that are not shown in the tables, the high NPY levels correlated with serum TNF-*α* levels (*r* = 0.32, *p* = 0.001), a decrease in BMI (*r* = −0.19, *p* = 0.04), and diminished fat mass (*r* = −0.23, *p* = 0.01). No correlations were observed between NPY levels and age, duration of RA, total bone mineral density, RF titles, and serum IL-6 or leptin concentrations. A trend of correlation between higher concentrations of anti-CCP2 and higher NPY levels (*r* = 0.17, *p* = 0.08) was observed, although this trend did not achieve statistical significance. TNF-*α* levels correlated with increased lean mass (*r* = 0.28, *p* = 0.003), low BMI (*r* = −0.24, *p* = 0.01), low fat mass (%) (*r* = −0.23, *p* = 0.01), and NPY levels (described above). TNF-*α* levels had a nonsignificant trend to correlate with long disease duration of RA (*r* = 0.17, *p* = 0.08), and a nonsignificant trend was observed between an increase in the TNF-*α* levels and a decrease in the glucocorticoid dosage (*r* = −0.18, *p* = 0.06). No significant correlations were observed between TNF-*α* levels and leptin, IL-6, RF, or anti-CCP antibodies. On the other hand, the serum concentrations of leptin correlated with higher fat mass (%) (*r* = 0.46, *p* < 0.001), higher dose of glucocorticoids (*r* = 0.25, *p* = 0.01), high functional disability according to the HAQ-DI score (*r* = 0.21, *p* = 0.05), and higher ESR (*r* = 0.27, *p* = 0.01). Finally, the serum levels of IL-6 correlated with higher fat mass (%) (*r* = 0.41, *p* < 0.001), but no correlations were observed with other variables.

Table 2 shows the comparisons of variables between RA patients in clinical remission (DAS28‐ESR < 2.6) and RA patients with active disease (DAS28‐ESR ≥ 2.6). Higher concentrations of NPY were observed in the group of patients with RA-active compared with those with RA in remission (*p* = 0.05). RA-active patients also have a higher disability according to the HAQ-DI score (*p* < 0.001), higher CRP levels (*p* = 0.001), higher ESR (*p* = 0.001), and elevated IL-6 levels (*p* = 0.04).

Table 3 shows the results of the logistic regression analysis assessing the variables associated with an increase in the risk of disease activity. After performing an adjustment by age, disease evolution, BMI, body fat mass (%), TNF-*α* levels, and use of biologic-DMARDs, we observed two variables that remain associated with an increased risk of disease activity: the serum NPY levels (OR: 1.04, 95% CI: 1.006-1.09, and *p* = 0.03) and the high functional disability by HAQ-DI (OR: 31.17, 95% CI: 5.90–164.67, and *p* < 0.001).

Table 4 shows the results of the multiple linear regression analysis. In this model, we tested the variables associated with NPY levels (used in this model as the dependent variable). After the adjustment by other variables, the serum TNF-*α* levels remained associated with NPY levels (*B* coefficient = 0.023, *p* = 0.008).

### 3.2. Discussion

In this study, we identified that the presence of disease activity in RA is associated with higher levels of NPY and that the serum levels of this neurotransmitter correlated positively with TNF-*α* levels, but it did not correlate with serum levels of leptin and IL-6. After adjusting by confounders, the increase in NPY levels remains associated with the presence of higher TNF-*α* levels and lower body fat mass. Using a multivariate logistic regression analysis after adjusting by potential confounders, NPY and high disability (HAQ-DI score) were both risk factors associated with disease activity.

Only a few studies have been conducted to evaluate the relationship between NPY levels and clinical characteristics in RA with nonconsistent results. However, to our knowledge, none of them has previously assessed the relation of NPY levels with disease activity and serum levels of TNF-*α* into the same study adjusting by other proinflammatory molecules. TNF-*α* is a proinflammatory cytokine that plays a protagonic role in the pathogenesis of the disease activity in RA patients. Some authors have identified that TNF-*α* increases the secretion of other proinflammatory cytokines and adipokines, including leptin [20]. Nevertheless, we did not observe any correlation between TNF-*α* levels and leptin or IL-6. Some authors have assessed a possible association between NPY and some clinical variables in RA patients treated with anti-TNF agents [12]. We examined in the present study a wide number of potential confounders of the relation between NPY levels and disease activity that were not previously investigated by other studies and identified an association between NPY levels and higher serum levels of TNF-*α*. Härle et al. noted that treating RA patients with anti-TNF agents decreased their NPY levels [11]. The mechanisms for explaining the effects of anti-TNF agents on NPY levels require further investigation. We hypothesized that NPY levels might decrease caused by the inhibition TNF-*α* activity on the cells that express NPY. The correlation between serum TNF-*α* concentrations and NPY levels observed by the present study might support the hypothesis of the role of TNF-*α* in increasing NPY, but this effect could also be secondary by other factors. Härle et al. identified that an increase in the fat mass accompanying the decrease of NPY was noticed in their patients after the use of TNF-*α* blockers [12]. TNF-*α* levels might also modify the synthesis of leptin inhibiting neuropeptide Y secretion [21], although in the present study, we did not observe any correlation between leptin and TNF-*α* levels or NPY levels. Therefore, TNF-*α* might produce an increase in leptin secretion in certain tissues, but not reflected on the circulant levels contributing to changes in body mass, and similarly an increase of NPY [21]. However, we must recognize as a limitation in the present study that leptin, interleukin 6, and TNF-*α* concentrations were not measured in all the patients with RA due to insufficient sera to quantify in all the samples these molecules. Nevertheless, the sample size was sufficient to demonstrate that the high NPY levels correlated with serum TNF-*α* levels. NPY concentrations did not correlate with other proinflammatory molecules, although we are confident that the statistical power was sufficient to establish conclusions with TNF-*α* levels; in the case of leptin and IL-6 correlations, we should be aware of the possibility of type II error in the results if there is no correlation with these two proinflammatory molecules.

NPY plays a crucial role in communication between the sympathetic nervous system (SNS) and the immune system [8]. However, the current function of NPY in the immune system in RA requires additional research. In murine models, NPY can inhibit cytokines produced by the T cells, decreasing some subpopulations of B cells and increasing subpopulations of naïve T cells [22]. Furthermore, NPY might have an effect on the leucocyte migration and adhesion to the endothelial wall [23]. On the other hand, in experimental studies, NPY can modulate the response of immune cells including macrophages, dendritic cells, neutrophils, or lymphocytes and induce the release of various proinflammatory cytokines including TNF-*α* and IL-6 and also interferon-gamma (IFN-*γ*) in activated macrophage [9].

We identified that the presence of disease activity is associated with higher levels of NPY. Similar to our results, Härle et al. observed a correlation between concentrations of NPY and DAS28-ESR [12]. Additionally, the results showed that high NPY levels associated simultaneously with disease activity and increases in TNF-*α* levels. However, Härle et al. did not find a correlation between NPY levels and DAS28-ESR and other inflammatory markers [11]. But these data should be considered in the light of an absence of a multivariate approach that adjusts the effect of potential confounders.

Our study is, to our knowledge, the first to evaluate the correlation between NPY levels and a varied spectrum of clinical variables including abnormalities on body composition, disease activity, TNF-*α*, IL-6, and leptin. Additionally, we performed different statistical models to evaluate the determinants of these variables using an adjusted analysis. We demonstrate that NPY levels are associated with TNF-*α*, and the clinical significance of this finding should be further explored in long-term follow-up studies to identify whether NPY levels might be considered a subrogated marker of disease activity in RA.

## 4. Conclusions

Serum levels of NPY are significantly related to TNF-*α* levels and disease activity in RA.

This study demonstrates that the NPY levels are associated with an increase of disease activity in RA independently of IL-6, TNF-*α*, or leptin levels. NPY levels can be considered a marker of activity of the disease. These results encourage future longitudinal studies to evaluate if higher NPY levels can be associated with the development other outcomes such as high disability and erosions in RA patients.

## Figures and Tables

**Table 1:** Clinical characteristics and body composition, in patients with rheumatoid arthritis.

Variables	*n* = 108
Age (years)	58.7 ± 11.1
RA disease duration (years)	13.9 ± 10.2
Clinical characteristics	
HAQ-DI score (units)	0.52 ± 0.58
Active disease (DAS28‐ESR ≥ 2.6), *n* (%)	68 (63)
DXA measurement of body composition	
Overweight or obesity, *n* (%)	76 (70.4)
Lean mass (%)	51.3 ± 5.5
Body fat mass > 33%, *n* (%)	105 (97.2)
Treatment	
Biologic-DMARDs, *n* (%)	13 (12)
Synthetic-DMARDs, *n* (%)	105 (97.2)
Methotrexate, *n* (%)	57 (52.8)
Sulfasalazine, *n* (%)	40 (37)
Leflunomide, *n* (%)	37 (34.3)
Azathioprine, *n* (%)	15 (13.9)
Chloroquine, *n* (%)	13 (12)
Corticosteroid users, *n* (%)	88 (81.5)
Laboratory variables	
C-reactive protein (mg/L)	16.8 ± 22.6
Erythrocyte sedimentation rate (mm/h)	29.2 ± 13.7
Rheumatoid factor (IU/mL), *n* = 73	230.05 ± 533.4
Positivity for rheumatoid factor (≥12 IU/mL), *n* (%)	57 (2.8)
Serum TNF-*α* levels (ng/mL), *n* = 106	37.9 ± 137.6
Serum IL-6 (pg/mL), *n* = 89	17.3 ± 38.3
Serum anti-CCP levels (RU/mL), *n* = 107	97.9 ± 125.5
Serum leptin levels (pg/ mL), *n* = 91	73.5 ± 62.9
Serum NPY levels (pg/mL), *n* = 108	20.9 ± 12.7

RA: rheumatoid arthritis; HAQ-DI: Health Assessment Questionnaire-Disability Index; DMARDs: disease-modifying antirheumatic drugs; TNF-*α*: tumor necrosis factor-alpha; IL-6: interleukin 6; NPY: neuropeptide Y.

**Table 2:** Comparison between the group with RA in remission (DAS28‐ESR < 2.6) and the group with RA-active disease (DAS28‐ESR ≥ 2.6).

Variable	RA in remission (DAS28‐ESR < 2.6) *n* = 40	RA-active (DAS28‐ESR ≥ 2.6) *n* = 68	*p*
Age (years)	56.6 ± 12.5	60 ± 10.01	0.12
RA disease duration (years)	12.1 ± 10.8	14.9 ± 9.7	0.16
DXA measurement of body composition			
Overweight and obesity, *n* (%)	33 (82.5)	43 (63.2)	0.05
Lean mass (%)	51.1 ± 5.2	51.5 ± 5.7	0.70
Body fat mass (%)	46.5 ± 5.2	46.7 ± 6.7	0.88
Treatment and clinical characteristics			
HAQ-DI score (units)	0.16 ± 0.22	0.72 ± 0.62	<0.001
Synthetic-DMARD use, *n* (%)	67 (98.5)	38 (95)	0.55
Biologic-DMARD use, *n* (%)	4 (10)	9 (13.2)	0.76
Corticosteroid dose (mg/day)	4.2 ± 2.9	5.2 ± 3.3	0.12
Laboratory variables			
CRP (mg/L)	8.07 ± 5.6	21.8 ± 26.9	0.001
ESR (mm/h)	24.1 ± 9.8	32.5 ± 14.8	0.001
RF (IU/mL)	112.3 ± 159.1	302.7 ± 659.1	0.06
TNF-*α* (ng/mL)	57.2 ± 209.5	27.2 ± 71.6	0.40
IL-6 (pg/mL)	8.6 ± 11.9	23.6 ± 48.3	0.04
Anti-CCP (RU/mL)	90.5 ± 112.05	102.3 ± 133.5	0.64
Leptin (pg/mL)	81.4 ± 65.5	67.9 ± 61.1	0.31
NPY (pg/mL)	17.8 ± 10.3	22.8 ± 13.6	0.05

RA: rheumatoid arthritis; DAS28-ESR: Disease Activity Score for 28 joints with Erythrocyte Sedimentation Rate; HAQ-DI: Health Assessment Questionnaire-Disability Index; DMARDs: disease-modifying antirheumatic drugs; TNF-*α*: tumor necrosis factor-alpha; IL-6: interleukin 6; NPY: neuropeptide Y. Comparisons between proportions were compared with the chi-squared test or Fisher's exact test (when required). Comparisons between means were evaluated with Student's *t*-test for independent samples.

**Table 3:** Results of the multivariate analysis evaluating factors associated with active disease in rheumatoid arthritis patients.

Risk factors of disease activity	Forward method (stepwise)		
	OR	95% CI	*p*
Serum NPY levels (pg/mL)	1.04	(1.006 to 1.09)	0.03
HAQ-DI score	31.17	(5.90-164.67)	<0.001

Logistic regression models using forward stepwise method. This model was adjusted by age, BMI, body fat mass, and biologic-DMARD use (anti-TNF-*α* agents). Dependent variable disease activity (active disease defined by DAS28 score ≥ 2.6 and remission DAS28 score of <2.6). OR: odds ratio; 95% CI: 95% statistical significance. *p* < 0.05.

**Table 4:** Factors associated with serum levels of NPY in rheumatoid arthritis (RA) in the multivariate multiple regression analysis.

	*B*	95% CI	*p*
Dependent variable: NPY levels			
TNF-*α* (pg/mL)	0.023	0.006 to 0.039	0.008

Models using forward stepwise method. The model was adjusted by age, BMI, anti-CCP levels, TNF-*α* levels, leptin levels, fat mass (%), and RA disease duration.

## Data Availability

The database used to support the findings of this study is available on request. If this database is required, please direct the correspondence to Dr. Laura Gonzalez-Lopez (dralauragonzalez@prodigy.net.mx) or Dr. Norma A. Rodriguez-Jimenez (azul_umi@hotmail.com).
